# The impact of levodopa on post-stroke depression: the ESTREL-depression-study

**DOI:** 10.1093/esj/aakag001

**Published:** 2026-02-17

**Authors:** Mirjam I Sauter, Josefin E Kaufmann, Lukas Boos, Annaelle Zietz, Simon Trüssel, Andreas R Luft, Alexandros Polymeris, Valerian L Altersberger, Karin Wiesner, Martina Wiegert, Jeremia P O Held, Yannik Rottenberger, Anne Schwarz, Friedrich Medlin, Ettore A Accolla, Sandrine Foucras, Georg Kägi, Gian Marco De Marchis, Svetlana Politz, Matthias Greulich, Alexander A Tarnutzer, Rolf Sturzenegger, Mira Katan, Marcel Arnold, Krassen Nedeltchev, Janine Schär, Katrien Van Den Keybus Deglon, Pierre-André Rapin, Alexander Salerno, David J Seiffge, Elias Auer, Julian Lippert, Leo H Bonati, Corina Schuster-Amft, Szabina Gäumann, Joelle N Chabwine, Andrea Humm, J Carsten Möller, Raoul Schweinfurther, Bartosz Bujan, Piotr Jedrysiak, Peter S Sandor, Roman Gonzenbach, Veit Mylius, Dietmar Lutz, Carmen Lienert, Nils Peters, Patrik Michel, René M Müri, Sabine Schädelin, Lars G Hemkens, Gary A Ford, Philippe A Lyrer, Henrik Gensicke, Christopher Traenka, Stefan T Engelter

**Affiliations:** Department of Rehabilitation and Neurology, University Department of Geriatric Medicine FELIX PLATTER, University of Basel, Basel, Switzerland; Department of Neurology and Stroke Center, Department of Clinical Research, University Hospital Basel and University of Basel, Basel, Switzerland; Department of Rehabilitation and Neurology, University Department of Geriatric Medicine FELIX PLATTER, University of Basel, Basel, Switzerland; Department of Neurology and Stroke Center, Department of Clinical Research, University Hospital Basel and University of Basel, Basel, Switzerland; Department of Rehabilitation and Neurology, University Department of Geriatric Medicine FELIX PLATTER, University of Basel, Basel, Switzerland; Department of Neurology and Stroke Center, Department of Clinical Research, University Hospital Basel and University of Basel, Basel, Switzerland; Department of Rehabilitation and Neurology, University Department of Geriatric Medicine FELIX PLATTER, University of Basel, Basel, Switzerland; Department of Neurology and Stroke Center, Department of Clinical Research, University Hospital Basel and University of Basel, Basel, Switzerland; Department of Rehabilitation and Neurology, University Department of Geriatric Medicine FELIX PLATTER, University of Basel, Basel, Switzerland; Department of Neurology and Stroke Center, Department of Clinical Research, University Hospital Basel and University of Basel, Basel, Switzerland; Division of Vascular Neurology and Neurorehabilitation, Department of Neurology, University Hospital of Zurich and University of Zurich, Zurich, Switzerland; Cereneo Center for Neurology and Rehabilitation, Vitznau, Switzerland; Department of Neurology and Stroke Center, Department of Clinical Research, University Hospital Basel and University of Basel, Basel, Switzerland; Stroke Division, Department of Neurology, Beth Israel Deaconess Medical Center, Harvard Medical School, Boston, MA, United States; Department of Neurology and Stroke Center, Department of Clinical Research, University Hospital Basel and University of Basel, Basel, Switzerland; Department of Medicine and Neurology, Melbourne Brain Centre at the Royal Melbourne Hospital, University of Melbourne, Parkville, VIC, Australia; Department of Rehabilitation and Neurology, University Department of Geriatric Medicine FELIX PLATTER, University of Basel, Basel, Switzerland; Department of Rehabilitation and Neurology, University Department of Geriatric Medicine FELIX PLATTER, University of Basel, Basel, Switzerland; Department of Neurology and Stroke Center, Department of Clinical Research, University Hospital Basel and University of Basel, Basel, Switzerland; Division of Vascular Neurology and Neurorehabilitation, Department of Neurology, University Hospital of Zurich and University of Zurich, Zurich, Switzerland; Bellevue Medical Group, Zurich, Switzerland; Division of Vascular Neurology and Neurorehabilitation, Department of Neurology, University Hospital of Zurich and University of Zurich, Zurich, Switzerland; Division of Vascular Neurology and Neurorehabilitation, Department of Neurology, University Hospital of Zurich and University of Zurich, Zurich, Switzerland; Department of Neurology, David Geffen School of Medicine at UCLA, Los Angeles, CA, United States; Service of Neurology and Stroke Unit, HFR Fribourg–Cantonal Hospital, Fribourg, Switzerland; Service of Neurology and Stroke Unit, HFR Fribourg–Cantonal Hospital, Fribourg, Switzerland; Service of Neurology and Stroke Unit, HFR Fribourg–Cantonal Hospital, Fribourg, Switzerland; University Teaching and Research Hospital, Neurology Department & Stroke Center, HOCH Health Ostschweiz, Kantonsspital St Gallen, St Gallen, Switzerland; Department of Neurology, Inselspital, Bern University Hospital and University of Bern, Bern, Switzerland; University Teaching and Research Hospital, Neurology Department & Stroke Center, HOCH Health Ostschweiz, Kantonsspital St Gallen, St Gallen, Switzerland; Kantonsspital Münsterlingen, Münsterlingen, Switzerland; Kantonsspital Winterthur, Winterthur, Switzerland; NeuroPraxis Zurich GmbH, Zurich, Switzerland; Neurology, Cantonal Hospital of Baden, Baden, Switzerland; Faculty of Medicine, University of Zurich, Zurich, Switzerland; Departement Innere Medizin, Neurologie, Kantonsspital Graubünden, Chur, Switzerland; Department of Neurology and Stroke Center, Department of Clinical Research, University Hospital Basel and University of Basel, Basel, Switzerland; Department of Neurology, University Hospital Bern, University of Bern, Bern, Switzerland; Cantonal Hospital of Aarau and Department of Neurology, University of Bern, Bern, Switzerland; Department of Neurology and Stroke Center, Klinik Hirslanden, Zurich, Switzerland; Acute Neurorehabilitation Unit, Department of Clinical Neurosciences, Lausanne University Hospital and University of Lausanne, Lausanne, Switzerland; Lavigny Rehabilitation Institution, Lavigny, Switzerland; Stroke Center, Service of Neurology, Department of Clinical Neurosciences, Lausanne University Hospital and University of Lausanne, Lausanne, Switzerland; Department of Neurology, University Hospital Bern, University of Bern, Bern, Switzerland; Department of Neurology, University Hospital Bern, University of Bern, Bern, Switzerland; Department of Neurology, University Hospital Bern, University of Bern, Bern, Switzerland; Department of Neurology and Stroke Center, Department of Clinical Research, University Hospital Basel and University of Basel, Basel, Switzerland; Research Department, Reha Rheinfelden, Rheinfelden, Switzerland; Research Department, Reha Rheinfelden, Rheinfelden, Switzerland; School of Engineering and Computer Science, Bern University of Applied Sciences, Biel, Switzerland; Department for Sport, Exercise and Health, University of Basel, Basel, Switzerland; Research Department, Reha Rheinfelden, Rheinfelden, Switzerland; Division of Neurorehabilitation, Fribourg Hospital, Meyriez-Murten, Switzerland; Laboratory for Neurorehabilitation Science, Medicine Section, University of Fribourg, Fribourg, Switzerland; Service of Neurology and Stroke Unit, HFR Fribourg–Cantonal Hospital, Fribourg, Switzerland; Division of Neurorehabilitation, Fribourg Hospital, Meyriez-Murten, Switzerland; Rehaklinik Zihlschlacht AG, Zihlschlacht-Sittendorf, Switzerland; Rehaklinik Zihlschlacht AG, Zihlschlacht-Sittendorf, Switzerland; Neurorehabilitation, Klinik Lengg Zurich, Zurich, Switzerland; Neurorehabilitation, Klinik Lengg Zurich, Zurich, Switzerland; Research Department, Rehaklinik Bad Zurzach, ZURZACH Care Group, Bad Zurzach, Switzerland; Kliniken Valens, St. Gallen, Switzerland; Kliniken Valens, St. Gallen, Switzerland; Kliniken Valens, St. Gallen, Switzerland; Kliniken Valens, St. Gallen, Switzerland; Department of Neurology and Stroke Center, Klinik Hirslanden, Zurich, Switzerland; Stroke Center, Service of Neurology, Department of Clinical Neurosciences, Lausanne University Hospital and University of Lausanne, Lausanne, Switzerland; Department of Neurology, Inselspital, Bern University Hospital and University of Bern, Bern, Switzerland; Gerontechnology and Rehabilitation Group, University of Bern, Bern, Switzerland; Department of Clinical Research, University Hospital Basel and University of Basel, Basel, Switzerland; Department of Clinical Research, University Hospital Basel and University of Basel, Basel, Switzerland; Pragmatic Evidence Lab, Research Center for Clinical Neuroimmunology and Neuroscience Basel, (RC2NB), University Hospital Basel and University of Basel, Basel, Switzerland; Meta-Research Innovation Center at Stanford (METRICS), Stanford University, Stanford, CA, United States; Radcliffe Department of Medicine, University of Oxford, Oxford, United Kingdom; Department of Neurology and Stroke Center, Department of Clinical Research, University Hospital Basel and University of Basel, Basel, Switzerland; Department of Rehabilitation and Neurology, University Department of Geriatric Medicine FELIX PLATTER, University of Basel, Basel, Switzerland; Department of Neurology and Stroke Center, Department of Clinical Research, University Hospital Basel and University of Basel, Basel, Switzerland; Department of Rehabilitation and Neurology, University Department of Geriatric Medicine FELIX PLATTER, University of Basel, Basel, Switzerland; Department of Neurology and Stroke Center, Department of Clinical Research, University Hospital Basel and University of Basel, Basel, Switzerland; Department of Rehabilitation and Neurology, University Department of Geriatric Medicine FELIX PLATTER, University of Basel, Basel, Switzerland; Department of Neurology and Stroke Center, Department of Clinical Research, University Hospital Basel and University of Basel, Basel, Switzerland

**Keywords:** depression, dopamine, levodopa, post-stroke depression, rehabilitation, stroke

## Abstract

**Introduction:**

Post-stroke depression (PSD) frequently occurs after acute stroke and negatively affects rehabilitation. Dopamine has beneficial effects on motivation and emotional stability. In stroke patients, low dopamine levels are linked to PSD. This study investigated whether levodopa treatment during in-hospital rehabilitation impacts PSD compared to placebo.

**Patients and methods:**

ESTREL-Depression was a pre-planned analysis of the multicenter, randomized, double-blind, placebo-controlled ESTREL trial. Participants with an acute ischemic or hemorrhagic stroke were randomly assigned to receive either levodopa/carbidopa (100/25 mg) or placebo three times daily for 39 days. All ESTREL participants with (1) information about the presence or absence of depression at three months and (2) who took at least 80% of the study medication were eligible for the study. Participants with a history of depression were excluded. For the primary outcome, the presence of PSD was defined as having a T-score of ≥55 in the Patient-Reported Outcomes Measurement Information System short-form depression-4a 3 months after randomization. Binary logistic regression was performed to assess the effect of levodopa on PSD.

**Results:**

The study included 407 ESTREL participants (median age 72, 60% male), 209 receiving levodopa, and 198 receiving placebo. At 3 months, the frequency and odds of PSD did not differ between the levodopa group (26%) and the placebo group (28%) (OR = 0.93, 95% CI, 0.60–1.43).

**Conclusion:**

In the ESTREL-Depression study, treatment with levodopa had no impact on the occurrence of PSD.

**Clinical Trial Registration:**

ClinicalTrials.gov: NCT03735901 (https://clinicaltrials.gov/study/NCT03735901).

## Graphical Abstract

**Graphical Abstract:**
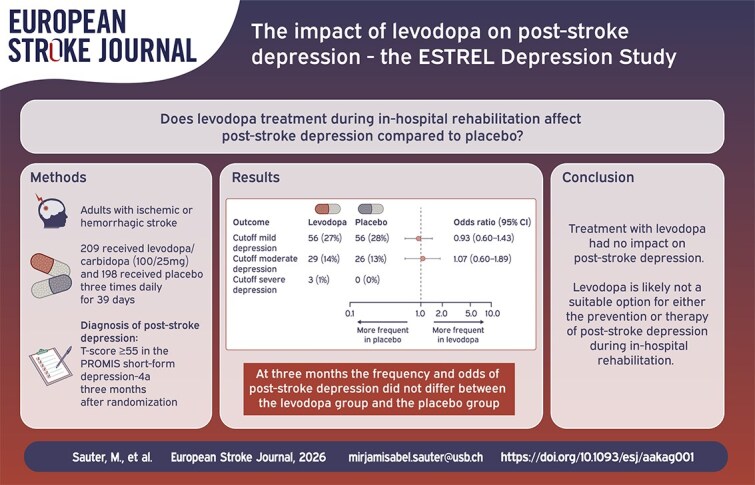

## Introduction

Post-stroke depression (PSD) affects approximately 25% of all stroke patients.^1^ PSD significantly hinders stroke recovery and rehabilitation.^2,3^ It is associated with poorer functional outcomes, increased mortality,^4^ cognitive deficits,^3^ greater long-term disability,^5^ and lower quality of life.^3^ Despite its relevance, PSD is often inadequately treated,^6^ and novel therapeutic options are clinically meaningful. A recent meta-analysis of PSD risk prediction models highlighted substantial methodological limitations, emphasizing the need for further research in PSD in a well-controlled setting.^7^

In non-stroke patients with depression, dopaminergic agents reportedly reduce depressive symptoms.^8^ However, there is limited evidence examining the effects of levodopa on depression.^9^ In patients with stroke, PSD is associated with low levels of monoamines, including dopamine.^10,11^ Reduced dopaminergic levels after stroke may occur secondarily due to stroke-related injury of dopaminergic pathways, rather than representing a primary etiological mechanism of PSD. In this scenario, enhancing dopaminergic signaling with levodopa could theoretically mitigate PSD by compensating for stroke-induced reductions in dopamine availability.^10^ In addition, dopamine exerts beneficial effects on motivation,^12^ emotional regulation, cognition, and neural plasticity.^13^ These observations suggest that levodopa, a precursor agent of dopamine, might have beneficial effects in reducing the risk of PSD during in-hospital rehabilitation, but hardly any clinical evidence exists.

Accordingly, this study aimed to investigate the impact of levodopa treatment on PSD during inpatient rehabilitation compared to placebo within the randomized controlled Enhancement of STroke REhabilitation with Levodopa (ESTREL) trial.

## Methods

### Study design

ESTREL-Depression was a pre-planned analysis of the ESTREL trial (NCT03735901). ESTREL investigated whether levodopa, compared to a placebo, enhanced motor recovery in patients following an acute stroke. ESTREL was a multicenter, randomized, parallel-group, double-blind, placebo-controlled trial across 13 acute stroke centers/units and 11 rehabilitation centers in Switzerland and recruited 610 participants between June 2019 and May 2024. Participants were randomly assigned in a 1:1 ratio to receive either levodopa/carbidopa 100/25 mg three times daily or a matching placebo for 39 days. The duration of the intervention was set to 39 days to reflect the usual length of inpatient stroke rehabilitation in Switzerland and to ensure the reliable intake of the study drug (ie, levodopa or placebo) during the controlled inpatient rehabilitation setting. This timeframe was predefined in the ESTREL protocol and chosen based on current concepts of post-stroke neuroplasticity, targeting the early rehabilitation phase.^14,15^ The study protocol was approved by all relevant ethics committees and legal authorities at participating sites.^14^ ESTREL enrolled previously independent patients (modified Rankin Scale (mRS) ≤ 3) with acute ischemic or hemorrhagic stroke and subsequent clinically meaningful hemiparesis. The complete inclusion and exclusion criteria of the ESTREL trial have been described previously.^14^ The primary outcome of ESTREL was the Fugl-Meyer Motor Assessment (FMMA) score after 3 months. The main results of the ESTREL trial have been published.^14,15^ In brief, ESTREL found no evidence that levodopa enhances motor recovery when added to standardized rehabilitation therapy.^15^

In order to study the potential impact on PSD, all ESTREL participants were asked to complete the Patient-Reported Outcomes Measurement Information System (PROMIS) form 29, which contains self-reported information about depression. All ESTREL participants who (1) provided information about the presence or absence of depression at three months and (2) took at least 80% of the study medication (ie, levodopa or placebo) were eligible for this analysis. Given the exploratory character of the study, the predefined primary analysis followed an on-treatment approach to assess the effect of levodopa. An intention-to-treat approach, in which all patients were analyzed, regardless of their actual study drug intake, was applied in the sensitivity analyses. Participants who had a history of depression were excluded to isolate PSD as the primary outcome of interest. A history of depression was identified based on documented clinical diagnoses in the medical records and discharge diagnosis lists prior to the index stroke.

### Participant characteristics

We used the following variables to characterize participants: age, sex, type of stroke (ie, ischemic versus hemorrhagic), affected arterial territory, affected brain hemisphere, time from index stroke to randomization, time from randomization to first administration of the study drug, use of antidepressant agents (Selective Serotonin Reuptake Inhibitors [SSRIs]/Serotonin-Norepinephrine Reuptake Inhibitors [SNRIs] or tricyclic antidepressants) and antiepileptic medication at baseline and after three months (recognizing that these agents may have been prescribed for indications other than depression, such as neuropathic pain), stroke characteristics (National Institutes of Health Stroke Scale (NIHSS), mRS, FMMA, and aphasia) at baseline and after 3 months.

### Primary outcomes

The primary outcome of ESTREL-Depression was the presence of depression at three months after randomization. Depression was assessed using the PROMIS short-form v1.0—depression 4a as a part of PROMIS 29 v1.2,^16,17^ a secondary outcome of ESTREL. This assessment consists of four questions, each scaled from 1 to 5. The answer options quantify in ascending order the frequency of symptoms from never (1 point) to rarely (2 points), to sometimes (3 points), to often (4 points), and to always (5 points) for each of the following feelings (1) worthlessness, (2) helplessness, (3) depression, and (4) hopelessness in the previous week.^17^ The total score ranges from 4 to 20 points. It is convertible into a T-score^17^ corresponding to the item response theory.^18^ A T-score of 50 corresponds to the US general population’s mean, and 10 corresponds to 1 SD.^19^ Since the depression score is negatively worded, a T-score of 60 is one SD more depressed than the mean of the US general population.^20^ For the primary outcome, the presence of PSD was defined with the cutoff for at least mild depression (T-score ≥ 55), as previously recommended.^16^

### Secondary outcomes

A secondary outcome was the severity of PSD, also using the T-score with the following categories: moderate depression (T-score ≥ 60, binary outcome), severe depression (T-score ≥ 70, binary outcome), and depression severity as an ordinal variable with four levels (no depression, mild depression, moderate depression, severe depression). This allowed us to compare (1) the frequency of moderate and severe depression and (2) the distribution of depression severity levels between both treatment groups. The PROMIS scales, along with their scoring manuals and instructions for using raw sum scores to T-score conversion tables, as well as the recommended T-score cutoffs, are available at www.healthmeasures.net.

### Statistical methods

The chi-squared test for categorical variables and the *t*-test for continuous variables were used to compare the distribution of participants’ characteristics between the levodopa and placebo groups (Table 1). Furthermore, the distribution of participants’ characteristics between the levodopa and placebo groups was compared, including participants with a history of depression (Table S1). An additional analysis was conducted to compare the characteristics of participants included in the ESTREL-Depression analysis with those excluded (Table S2). Only participants with complete PROMIS short-form depression-4a data at three months were included in the analyses. No imputation for missing outcome data was performed, corresponding to a complete-case analysis.

**Table 1:** Participant characteristics at baseline and three months after randomization by allocated treatment.

	**Overall**	**Levodopa**	**Placebo**	***P***
***n***	407	209	198	
**Age**, median [IQR]	72 [63, 82]	72 [62, 81]	74 [64, 83]	.08
**Sex**				
Male, *n* (%)	246 (60.4)	134 (64.1)	112 (56.6)	.15
Female, *n* (%)	161 (39.6)	75 (35.9)	86 (43.4)	.15
**Type of stroke**				
Acute ischemic stroke, *n* (%)	350 (86.0)	180 (86.1)	170 (85.9)	1.00
Acute hemorrhagic stroke, *n* (%)	57 (14.0)	29 (13.9)	28 (14.1)	1.00
**Affected arterial territory**				
Middle cerebral artery, *n* (%)	311 (76.4)	159 (76.1)	152 (76.8)	.96
Anterior cerebral artery, *n* (%)	41 (10.1)	26 (12.4)	15 (7.6)	.14
Posterior cerebral artery, *n* (%)	28 (6.9)	14 (6.7)	14 (7.1)	1.00
Vertebrobasilar arteries, *n* (%)	73 (17.9)	39 (18.7)	34 (17.2)	.79
**Affected brain hemisphere**				
Left, *n* (%)	161 (39.6)	83 (39.7)	78 (39.4)	1.00
Right, *n* (%)	263 (64.6)	137 (65.6)	126 (63.6)	.76
Bilateral, *n* (%)	17 (4.2)	11 (5.3)	6 (3.0)	.38
**Days from index stroke to randomization**, median [IQR]	3.00 [2, 5]	3.00 [2, 5]	3.00 [2, 5]	.801
**Days from randomization to first study drug administration**, median [IQR]	1.00 [1, 1]	1.00 [1, 1]	1.00 [1, 1]	.436
**Antidepressant agents at baseline**, *n* (%)	36 (8.8)	18 (8.6)	18 (9.1)	1.00
SSRI/SNRI, *n* (%)	30 (7.4)	14 (6.7)	16 (8.1)	.73
Tricyclic antidepressant, *n* (%)	7 (1.7)	4 (1.9)	3 (1.5)	1.00
**Antidepressant agents after three months**, *n* (%)	107 (26.3)	63 (30.1)	44 (22.2)	.09
SSRI/SNRI, *n* (%)	96 (23.6)	57 (27.3)	39 (19.7)	.09
Tricyclic antidepressant, *n* (%)	15 (3.7)	8 (3.8)	7 (3.5)	1.00
**Antiepileptic drugs**				
At baseline, *n* (%)	27 (6.6)	10 (4.8)	17 (8.6)	.180
After 3 months, *n* (%)	56 (13.8)	24 (11.5)	32 (16.2)	.220
**Stroke characteristics at baseline**				
NIHSS, median [IQR]	7 [5, 10]	7 [5, 10]	7 [5, 10]	.59
mRS, median [IQR]	4 [4, 5]	4 [4, 4]	4 [4, 5]	.21
FMMA, median [IQR]^a^	37 [17, 58]	38 [18, 59]	36 [17, 57]	.73
Aphasia, *n* (%)	59 (14.5)	36 (17.2)	23 (11.6)	.14
**Stroke characteristics after three months**				
NIHSS, median [IQR]^b^	3 [1, 5]	3 [1, 5.25]	3 [2, 5]	.49
mRS, median [IQR]	3 [2, 4]	3 [2, 4]	3 [2, 4]	.91
FMMA, median [IQR]^b^	70 [45, 86]	71 [44, 87]	67 [49, 84]	.88
Aphasia, n (%)^b^	39 (9.6)	22 (10.6)	17 (8.6)	.62
**Adverse events**, *n* (%)	85 (20.9)	51 (24.4)	34 (17.2)	.10
Reported depression, *n* (%)	10 (2.5)	5 (2.4)	5 (2.5)	1.00
**Severe adverse events**, *n* (%)	121 (29.7)	58 (27.8)	63 (31.8)	.43

^a^Data missing for one participant.

^b^Data missing for two participants.

Data expressed as median with Interquartile Range (IQR) and number (%) representing the data.

Abbreviations: FMMA = Fugl-Meyer motor assessment; mRS = modified rankin scale; NIHSS = National Institutes of Health stroke scale; SNRI = serotonin noradrenalin reuptake inhibitor; SSRI = selective serotonin reuptake inhibitor.

Unadjusted binary logistic regression was used to compare the odds of depression between the levodopa and placebo groups for the primary outcome at three months. The results are presented in forest plots as odds ratios (ORs) with corresponding 95% CIs (Figure 2).

**Figure 1:**
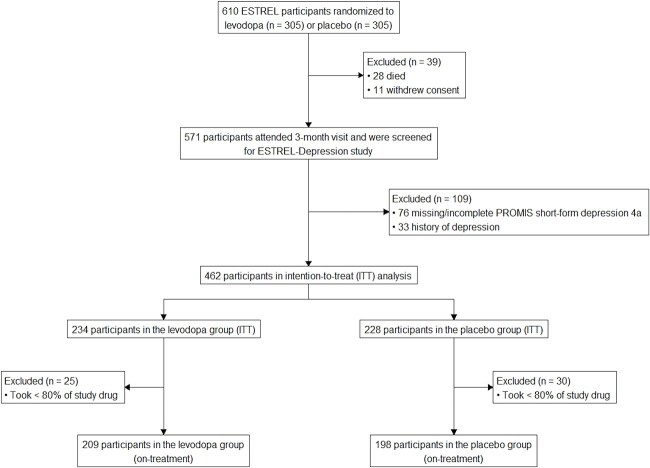
Study flow chart. Abbreviations: ITT = intention-to-treat, on-treatment analysis = analysis including the participants who took less than 80% of the study drug.

**Figure 2:**
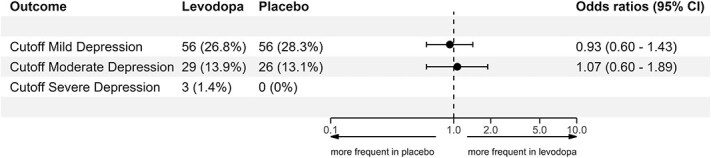
Forest plot of primary outcome and depression severity. The proportion of depression was measured using different cutoff T-scores of the PROMIS short-form depression-4a at three months, showing the number and proportion of participants for each levodopa and placebo group. Cutoff mild depression = T-score ≥ 55, cutoff moderate depression = T-score ≥ 60, cutoff severe depression = T-score ≥ 70.

**Figure 3:**
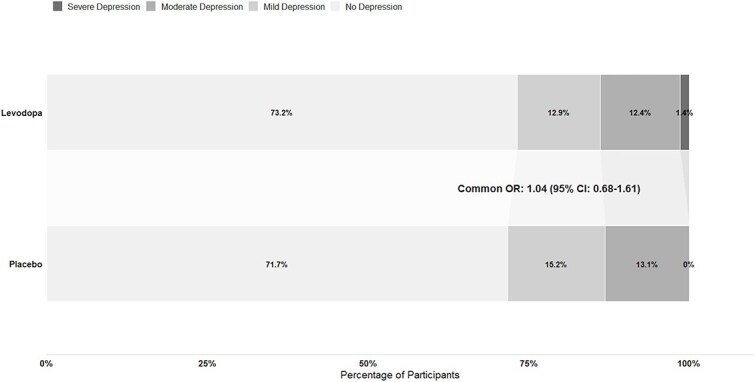
Distribution of the proportion of participants with different levels of depression severity in both treatment groups at 3 months. No depression = T-score < 55, mild depression = T-score ≥ 55 and < 60, moderate depression = T-score ≥ 60 and < 70, severe depression = T-score ≥ 70.

**Figure 4:**
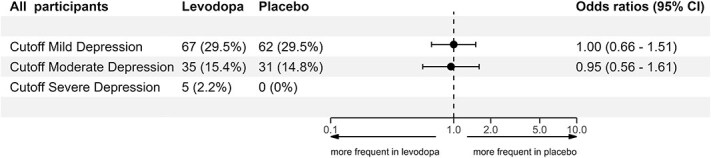
Sensitivity analysis including all participants regardless of their history of depression. Exploring the proportion of depression using different cutoff T-scores of the PROMIS short-form depression-4a at three months with the number and proportion of participants each for the levodopa and placebo group. Cutoff mild depression = T-score ≥ 55, cutoff moderate depression = T-score ≥ 60, cutoff severe depression = T-score ≥ 70.

To compare the distribution of depression severity levels between the levodopa and placebo groups, we performed an ordinal shift analysis (Figure 3). As sensitivity analyses, we repeated the primary outcome analysis, including participants who had a history of depression and had been excluded from the primary analysis (Figure 4). As an additional post hoc sensitivity analysis, we compared the on-treatment results with an intention-to-treat approach across different PROMIS depression severity cutoffs (Table S3).

To assess the potential confounding effect of antidepressant use, post hoc sensitivity analyses were performed by adjusting the primary analysis for antidepressant intake and stratifying analyses by any use and no use of antidepressants during follow-up (Figures S1-S3).

**Figure 5:**
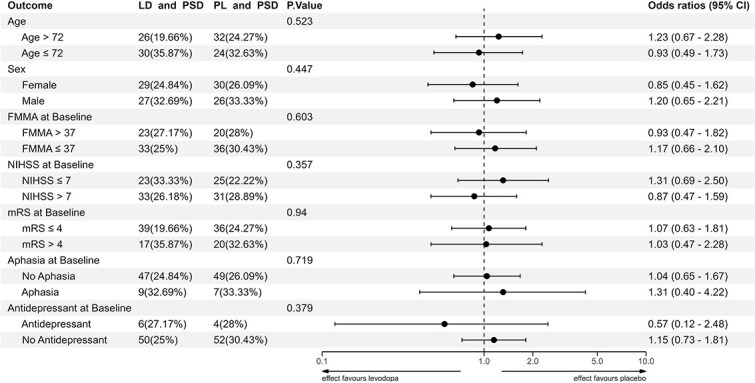
Interaction analysis of participant characteristics and the impact of levodopa on PSD. This interaction analysis shows how participant baseline characteristics are associated with the impact of levodopa on depression three months post-randomization. The median was chosen to convert each continuous numerical variable into a binary format. Abbreviations: FMMA = Fugl-Meyer Motor; LD = levodopa; mRS = modified rankin scale; NIHSS = National Institutes of Health Stroke Scale; PL = placebo.

Furthermore, we conducted exploratory logistic regression interaction models using the participant characteristics described above to assess whether the impact of levodopa on depression depends on the participants’ baseline characteristics (Figure 5). Associations between the presence of PSD and participant characteristics at baseline (Figure S4) and at three months (Figure S5) were assessed using univariate binary logistic regression analysis for each characteristic. The median was chosen to convert each continuous variable into a binary format. We also performed a chi-squared test to compare the use of antidepressants between the levodopa and placebo groups at baseline, at five weeks, and at three months, which we visualized in a histogram (Figure S6).

A *P*-value of 0.05 or less determined statistical significance. The statistics were calculated with R version 4.4.1 (R Core Team, 2024).

## Results

### Study population

Of the 610 ESTREL participants, 28 died, and 11 withdrew consent before the three-month visit. The remaining 571 participants were screened for inclusion in the ESTREL-Depression analysis. Among these, 76 were excluded due to missing or incomplete PROMIS short-form depression-4a data, and 33 were excluded because of a history of depression. This resulted in 462 participants eligible for the intention-to-treat analysis (234 in the levodopa group and 228 in the placebo group). An additional 55 participants were excluded for taking <80% of the study medication, leaving 407 participants for the on-treatment analysis (209 receiving levodopa and 198 receiving placebo).

### Participant characteristics

Participants had a median age of 72 years; 60% were male, and 86% of strokes were ischemic. At baseline, the mean NIHSS was 7, and 14.5% had aphasia. At three months, the mean T-score over all participants was 49.4 using the PROMIS short-form depression-4a. Antiepileptic drugs were used by 27 participants (6.6%) at baseline and by 56 participants (13.8%) at 3 months, without significant differences between the levodopa and placebo groups. The characteristics of the levodopa and placebo groups were well-balanced across all measured variables (Table 1). Table S1 shows the distribution of participant characteristics across the two treatment arms, including participants with a history of depression. Furthermore, Table S2 presents the participant characteristics for all ESTREL participants, those included in the ESTREL-Depression-Study, and those excluded.

### Primary outcome

At the three-month assessment, 56/209 (27%) participants in the levodopa group had PSD compared to 56/198 (28%) participants in the placebo group, amounting to an OR of 0.93 (95% CI, 0.60–1.43).

### Secondary outcomes

Using different PSD severity levels, the results showed that at least moderate severity of PSD occurred in 29/209 (14%) participants treated with levodopa, compared to 26/198 (13%) treated with placebo (OR 1.07; 95% CI, 0.60–1.89). Severe PSD occurred only in three participants in the levodopa group and none in the placebo group (Figure 2).

The distribution of PSD severity did not differ between the levodopa and placebo groups (common OR = 1.04; 95% CI, 0.68–1.61, Figure 3).

### Sensitivity analyses

The sensitivity analysis, including an additional 30 participants with a history of depression, was consistent with the primary outcome findings. 67/227 (30%) levodopa-treated participants had PSD, compared to 62/210 (30%) placebo participants (OR 1.00; 95% CI, 0.66–1.51) (Figure 4). Results were similar when comparing on-treatment and intention-to-treat analyses across the different PROMIS depression severity cutoffs (Table S3). Additional sensitivity analyses adjusting the primary outcome for antidepressant use, handled as a covariate in the logistic regression model, showed results consistent with the main analysis (Figure S1). Likewise, stratified analyses restricted to participants with antidepressant use (Figure S2) and to those without antidepressant use (Figure S3) did not reveal a differential effect of levodopa on PSD. Furthermore, the use of antidepressant agents did not differ significantly between the groups at baseline (9% in both), at five weeks (levodopa 30%, placebo 24%), or three months (levodopa 30%, placebo 22%) (Figure S6).

### Interaction analysis

The effect of levodopa on depression did not differ across examined subgroups, including age, sex, baseline FMMA, NIHSS, mRS, aphasia, and antidepressant use (Figure 5).

### Associations between PSD and participant characteristics

Occurrence of PSD was associated with female sex (OR = 2.11, 95% CI, 1.36–3.28), lower FMMA (≤37) at baseline (OR = 1.84, 95% CI, 1.18–2.88), and higher NIHSS (>7) at baseline (OR = 1.86, 95% CI, 1.20–2.91) (Figure S4). After three months, PSD was linked to impaired functional outcomes (FMMA ≤ 70, OR = 1.98, 95% CI, 1.27–3.11; NIHSS > 3, OR = 1.75, 95% CI, 1.13–2.72; mRS > 3, OR = 3.06, 95% CI, 1.93–4.85), but probably not to aphasia (OR = 1.53, 95% CI, 0.75–3.03) (Figure S5).

## Discussion

In this sub-study of the ESTREL trial, we found that treatment with levodopa had no impact on the occurrence and severity of PSD three months after the index stroke.

Despite the role of dopamine in mood regulation^21^ and the association of lower dopamine levels with PSD,^3^ our findings do not indicate an effect of levodopa on PSD. In a randomized controlled trial in 1997 among 21 stroke patients, participants treated with methylphenidate (≈22 mg/day, ≤3 weeks) had a lower depression score during post-stroke recovery than those treated with placebo.^22^ In addition, in non-stroke patients, systematic reviews suggest dopaminergic agents (eg, pramipexole, modafinil/armodafinil, stimulants) as a treatment option for bipolar depression^8^ and as an augmentation in treatment-resistant major depression.^23^ However, these reports provided no information on the effect of levodopa on those patients. Another randomized controlled trial reported a reduction in PSD among participants receiving combined dopaminergic treatment of methylphenidate (20 mg/day) and levodopa (125 mg/day) for 15 days, compared to the placebo group,^24^ while only minor effects were reported for the groups receiving either drug alone (20 mg methylphenidate/day or 125 mg Levodopa/day for 15 days).^24^ The relatively small population size of 20 participants in the levodopa-only group limits the interpretation of these findings. Interestingly, the levodopa dosage was lower and the treatment duration shorter^24^ than in our study (125 mg/day for 15 days vs. 3 × 125 mg/day for 39 days in ESTREL. The dosage used in ESTREL is similar to studies focused on enhanced learning in healthy humans^14,25,26^ and RCTs regarding stroke motor recovery,^14,27-29^ which administered levodopa at doses of 100 mg per administration over varying treatment durations.^24-26,28,29^ Our observed absence of a levodopa effect on PSD suggests that dopaminergic augmentation alone may be insufficient to treat PSD. Given the limited effect of levodopa in this study, future interventions should consider combined pharmacological approaches or individualized treatment strategies that target multiple mechanisms to enhance efficacy in treating PSD.

Several mechanistic explanations may account for the absence of a levodopa effect on PSD in our study. First, dopaminergic dysfunction after stroke may arise as a secondary consequence of structural brain injury rather than representing a primary etiological driver of PSD.^10,30^ Experiments in mice had shown that focal ischemic injury can lead to secondary degeneration of dopaminergic neurons and reduced striatal dopamine levels, suggesting that dopaminergic alterations may reflect downstream effects of stroke pathology rather than a direct causal mechanism of depression.^10,30^ If dopaminergic alterations primarily reflect irreversible downstream effects of stroke pathology, rather than a modifiable mechanism of depression, levodopa may be insufficient to meaningfully influence PSD risk, which could partly explain the absence of an observed treatment effect. Second, PSD is increasingly recognized as a multifactorial condition involving alterations in monoaminergic, inflammatory pathways, neural network changes, as well as genetic variations and psychosocial factors, which may limit the therapeutic impact of isolated dopaminergic augmentation.^10^

In ESTREL-depression, the prevalence of PSD was 28% at 3 months, which is higher than the rates reported in the pivotal randomized controlled trials (ie, EFFECTS, AFFINITY, and FOCUS) that compared the effect of SSRI versus placebo on PSD, ranging from 10 to 17%.^31-33^ Participants in these trials had less severe strokes (median NIHSS of 3-6),^31-34^ compared to ours (median NIHSS of 7). This difference may contribute to the higher prevalence of PSD observed in our study, as stroke severity is known to be associated with PSD.^3^ Additionally, differences in depression assessment tools and evaluation time points may further explain the variation in reported prevalence rates of PSD. Interestingly, the PSD prevalence in our study aligns well with Liu et al.’s meta-analysis about the prevalence and natural history of depression after stroke, which estimated a prevalence of 27% at any time post-stroke and within one to five months after stroke.^1^

Although many participants started therapy with antidepressants during rehabilitation, their distribution was similar between the treatment arms, and multiple sensitivity analyses indicated that antidepressant use did not confound the association between levodopa and PSD.

### Limitations

We are aware of important limitations. The main limitation of this study is the use of a short general depression screening scale rather than a specialized diagnostic instrument, such as the Hospital Anxiety and Depression Scale Depression subscale^35^ or the PHQ-9.^36^ While the widely used PHQ-9 includes the affective, cognitive, and somatic aspects of depression as defined in the DSM,^20^ the PROMIS scale focuses primarily on the affective aspects of depression.^20^ However, Kroenke et al. showed that the PROMIS short-form depression-4a has a diagnostic accuracy for depression comparable to the PHQ-9.^20,37^ Although PROMIS short-form depression-4a and the cutoff T-score ≥ 55 are supported by prior recommendations,^20,38^ validation in post-stroke populations remains limited.

Another limitation is a selection bias, as patients who did not complete the PROMIS questionnaire may have differed systematically from those who did. A further limitation is that the use of a self-reported PROMIS scale may have led to an underestimation of PSD in participants with cognitive or language deficits. Although severe aphasia was an exclusion criterion, milder impairments may still have affected the results. Additionally, the presence or absence of depression was not assessed at baseline, limiting insight into changes over time. The presence of other psychiatric disorders, including substance abuse, was not systematically assessed, which may have influenced the occurrence of mood disorders, including PSD. Moreover, the lack of detailed information on the specific indications for the prescription of antidepressant agents and antiepileptic drugs limits our ability to determine whether, and how these agents may have influenced PSD occurrence or severity.^39^

Furthermore, our findings are based on Swiss participants, which may limit their generalizability to other populations. Since we had no information on the participants’ education levels, we were unable to conduct sub-analyses on their influence on PSD. In addition, due to our study design, we were unable to examine the impact of levodopa on PSD using higher doses, longer treatment durations, combination therapies, or targeted approaches, such as trials in anatomically defined subgroups or the use of neuroimaging to identify dopaminergic deficits. We did not assess whether stroke lesions involved dopaminergic subcortical structures, which may be relevant for interpreting the potential effects of dopaminergic therapy on PSD. These strategies could help refine patient selection and provide deeper insights into the potential role of dopaminergic therapy in PSD.

### Strengths

This study had several strengths. It is based on a large dataset from a randomized controlled trial, which provided a robust sample size with minimal missing data. Standardized procedures and the availability of monitored core data further enhance its reliability. Furthermore, our patient population showed characteristics consistent with most known PSD-related factors.^3,40^ Further, our mean T-score of 49.4 aligns with a prior study using the PROMIS short-form depression-4a, which reported a mean of 51.3 three months post-stroke.^37^ Furthermore, the PROMIS short-form depression-4a is a suitable option for depression screening,^20,37^ with a T-score of 55 confirmed as an optimal cutoff for depression screening.^16,20^ In addition, the results were robust across multiple sensitivity analyses, including an intention-to-treat approach, inclusion of participants with a history of depression, and analyses using alternative PROMIS cutoffs for diagnosing PSD.

## Conclusion

In the ESTREL-Depression study, PSD was common among stroke survivors with motor deficits. Treatment with levodopa following an acute stroke had no impact on PSD. These findings suggest that levodopa is unlikely to be a suitable treatment option for PSD during in-hospital rehabilitation.

## Supplementary Material

aakag001_Supplementary_Figures_and_Tables_Revision_clean

aakag001_Supplement_2_Nonauthor_Collaborators

## Data Availability

Trial data can be made available on reasonable request to the corresponding author. Such requests must be accompanied by detailed study proposals, a description of study objectives, and a statistical analysis plan. Additional material might be requested during the assessments of the study eligibility for data sharing, which must be approved by the corresponding author, the sponsor-investigator of the trial, the trial steering committee, and the principal investigators of each center. Each request will be checked for compatibility with regulatory (ethics committee) requirements and with patient-informed consent.

## Patient and public involvement

Patients or members of the public were not involved in this study’s design, conduct, or recruitment. The research question and outcome measures were based on established clinical priorities and existing scientific evidence.

## Publishing rights statement

The corresponding author has the right to grant on behalf of all authors and does grant on behalf of all authors, a worldwide license to the Publishers and its licenses in perpetuity, in all forms, formats and media (whether known now or created in the future), to (1) publish, reproduce, distribute, display and store the Contribution, (2) translate the Contribution into other languages, create adaptations, reprints, include within collections and create summaries, extracts and/or, abstracts of the Contribution, (3) create any other derivative work(s) based on the Contribution, (4) to exploit all subsidiary rights in the Contribution, (5) the inclusion of electronic links from the Contribution to third party material where-ever it may be located; and, (6) license any third party to do any or all of the above.
