# Supplementary material for: The impact of levodopa on post-stroke depression: the ESTREL-depression-study
Source: Eur Stroke J. 2026 Feb 17;11(2):aakag001. doi: 10.1093/esj/aakag001 (PMC12911922; doi:10.1093/esj/aakag001)
Supplement: aakag001_Supplementary_Figures_and_Tables_Revision_clean [file aakag001_supplementary_figures_and_tables_revision_clean.docx]

Supplementary Materials for the ESTREL-Depression-Study

Supplementary Tables

[Table S1: Participant characteristics according to allocated treatment, including the participants with a history of depression. 2](#_Toc216331863)

[Table S2: Comparison of participant characteristics between all ESTREL participants, ESTREL-Depression participants, and excluded participants for ESTREL-Depression. 3](#_Toc216331864)

[Table S3: Sensitivity analyses comparing on-treatment and intention-to-treat estimates across depression severity cutoffs. 4](#_Toc216331865)

Supplementary Figures

[Figure S1: Sensitivity analysis adjusting the primary outcome for antidepressant use. 5](#_Toc216331902)

[Figure S2: Sensitivity analysis restricted to participants with antidepressant use during follow-up. 6](#_Toc216331903)

[Figure S3: Sensitivity analysis restricted to participants without antidepressant use during follow-up. 7](#_Toc216331904)

[Figure S4: Associations between PSD and participant characteristics. 8](#_Toc216331905)

[Figure S5: Association between outcomes after three months and PSD. 9](#_Toc216331906)

[Figure S6: Use of antidepressants at baseline and during follow-up. 10](#_Toc216331907)

**Table S1: Participant characteristics according to allocated treatment, including the participants with a history of depression.**

|  | **Overall** | **Levodopa** | **Placebo** | **p** |
| --- | --- | --- | --- | --- |
| **n** | 437 | 227 | 210 |  |
| **Age**, median [IQR] | 72 [62, 82] | 72 [62, 81] | 74 [63, 82] | 0.14 |
| **Sex** |  |  |  |  |
| Male, n (%) | 259 (59.3) | 143 (63.0) | 116 (55.2) | 0.12 |
| Female, n (%) | 178 (40.7) | 84 (37.0) | 94 (44.8) | 0.12 |
| **Type of stroke** |  |  |  |  |
| Acute ischemic stroke, n (%) | 375 (85.8) | 196 (86.3) | 179 (85.2) | 0.85 |
| Acute hemorrhagic stroke, n (%) | 62 (14.2) | 31 (13.7) | 31 (14.8) | 0.85 |
| **Affected arterial territory** |  |  |  |  |
| Middle cerebral artery, n (%) | 330 (75.5) | 168 (74.0) | 162 (77.1) | 0.52 |
| Anterior cerebral artery, n (%) | 43 (9.8) | 28 (12.3) | 15 (7.1) | 0.10 |
| Posterior cerebral artery, n (%) | 32 (7.3) | 16 (7.0) | 16 (7.6) | 0.10 |
| Vertebrobasilar arteries, n (%) | 81 (18.5) | 46 (20.3) | 35 (16.7) | 0.40 |
| **Affected brain hemisphere** |  |  |  |  |
| Left, n (%) | 188 (43.0) | 94 (41.4) | 94 (44.8) | 0.54 |
| Right, n (%) | 244 (55.8) | 128 (56.4) | 116 (55.2) | 0.88 |
| Bilateral, n (%) | 25 (5.7) | 14 (6.2) | 11 (5.2) | 0.83 |
| **History of depression**, n (%) | 30 (6.9) | 18 (7.9) | 12 (5.7) | 0.47 |
| **Antidepressant agents at baseline**, n (%) | 59 (13.5) | 35 (15.4) | 24 (11.4) | 0.28 |
| SSRI/SNRI, n (%) | 51 (11.7) | 29 (12.8) | 22 (10.5) | 0.55 |
| Tricyclic antidepressant, n (%) | 10 (2.3) | 6 (2.6) | 4 (1.9) | 0.85 |
| **Antidepressant agents after three months**, n (%) | 132 (30.2) | 79 (34.8) | 53 (25.2) | 0.04 |
| SSRI/SNRI, n (%) | 119 (27.2) | 71 (31.3) | 48 (22.9) | 0.06 |
| Tricyclic antidepressant, n (%) | 17 (3.9) | 10 (4.4) | 7 (3.3) | 0.74 |
| **Stroke characteristics at baseline** |  |  |  |  |
| NIHSS, median [IQR] | 7 [5, 10] | 7 [5, 10] | 7 [5, 10] | 0.65 |
| mRS, median [IQR] | 4 [4, 5] | 4 [4, 5] | 4 [4, 5] | 0.45 |
| FMMA, median [IQR] * | 36 [17, 58] | 37 [17, 58] | 35 [16, 57] | 0.87 |
| Aphasia at baseline, n (%) * | 63 (14.4) | 39 (17.2) | 24 (11.5) | 0.12 |
| **Stroke characteristics after three months** |  |  |  |  |
| NIHSS, median [IQR] † | 3 [2, 5] | 3 [1, 6] | 3 [2, 5] | 0.78 |
| mRS, median [IQR] | 3 [2, 4] | 3 [2, 4] | 3 [2, 4] | 0.52 |
| FMMA, median [IQR] † | 70 [46, 85] | 70 [44, 87] | 70 [49, 84] | 0.86 |
| Aphasia, n (%) † | 41 (9.4) | 23 (10.2) | 18 (8.6) | 0.69 |

*Data expressed as median with Interquartile Range (IQR) and number (%) representing the data.*

*SSRI = Selective Serotonin Reuptake Inhibitor, SNRI = Serotonin Noradrenalin Reuptake Inhibitor,* *NIHSS = National Institutes of Health Stroke Scale, mRS = modified Rankin Scale, FMMA = Fugl-Meyer Motor Assessment.*

** Data missing for one participant.*

*† Data missing for two participants.*

**Table S2: Comparison of participant characteristics between all ESTREL participants, ESTREL-Depression participants, and excluded participants for ESTREL-Depression.**

|  | **Overall** | **Excluded** | **Included** | **p** |
| --- | --- | --- | --- | --- |
| **n** | 610 | 203 | 407 |  |
| **Age**, median [IQR] | 73 [64, 82] | 75 [65, 82] | 72 [63, 82] | 0.12 |
| **Sex** |  |  |  |  |
| Male, n (%) | 358 (58.7) | 112 (55.2) | 246 (60.4) | 0.25 |
| Female, n (%) | 252 (41.3) | 91 (44.8) | 161 (39.6) | 0.25 |
| **Antidepressant agents at baseline**, n (%) | 84 (13.8) | 48 (23.6) | 36 (8.8) | <0.001 |
| **Antidepressant agents after three months**, n (%) | 178 (31.1) | 71 (43.0) | 107 (26.3) | <0.001 |
| **Stroke characteristics at baseline** |  |  |  |  |
| NIHSS, median [IQR] | 7 [5, 10] | 8.00 [5, 11] | 7.00 [5, 10] | 0.002 |
| mRS, median [IQR] | 4.00 [4, 5] | 4.00 [4, 5] | 4.00 [4, 5] | 0.06 |
| FMMA, median [IQR] | 34 [14, 54] | 24 [9, 46] | 37 [17, 58] | <0.001 |
| Aphasia, n (%) | 96 (15.9) | 37 (18.7) | 59 (14.5) | 0.23 |
| **Stroke characteristics after three months** |  |  |  |  |
| NIHSS, median [IQR] | 3 [2, 6] | 4 [2, 7] | 3 [1, 5] | 0.01 |
| mRS, median [IQR] | 3 [2, 4] | 3 [2, 4] | 3 [2, 4] | <0.001 |
| FMMA, median [IQR] | 68 [42, 85] | 61 [34, 82] | 70 [45, 86] | 0.07 |
| Aphasia, n (%) | 63 (11.4) | 24 (16.4) | 39 (9.6) | 0.04 |
| **Score at Depression shortform-4a in PROMIS 29**, median [IQR] | 5 [4, 8] | 6 [4, 9] | 5 [4, 8] | 0.07 |
| **History of Depression**, n (%) | 41 (6.7) | 41 (20.2) | 0 (0.0) | <0.001 |

*Data expressed as median with Interquartile Range (IQR) and number (%) representing the data.*

*NIHSS = National Institutes of Health Stroke Scale, mRS = modified Rankin Scale, FMMA = Fugl-Meyer Motor Assessment, PROMIS = Patient-Reported Outcomes Measurement Information System.*

**Table S3: Sensitivity analyses comparing on-treatment and intention-to-treat estimates across depression severity cutoffs.**

| **Outcome** | **On-treatment Estimate** | **Intention to treat** |
| --- | --- | --- |
| **Cutoff Mild Depression** | OR 0.93 (95% CI 0.60-1.43) | OR 0.87 (95% CI 0.58-1.30) |
| **Cutoff Moderate Depression** | OR 1.07 (95% CI 0.60-1.89) | OR 1.05 (95% CI 0.61-1.79) |
| **Cutoff Severe Depression** | **-** | **-** |

*Cutoff mild depression = T-score ≥55, cutoff moderate depression = T-score ≥60, cutoff severe depression = T-score ≥70.*

**Figure S1: Sensitivity analysis adjusting the primary outcome for antidepressant use.**

The proportion of depression at three months is shown using different cutoff T-scores of the PROMIS short-form depression-4a, with the number and proportion of participants in the levodopa and placebo groups.


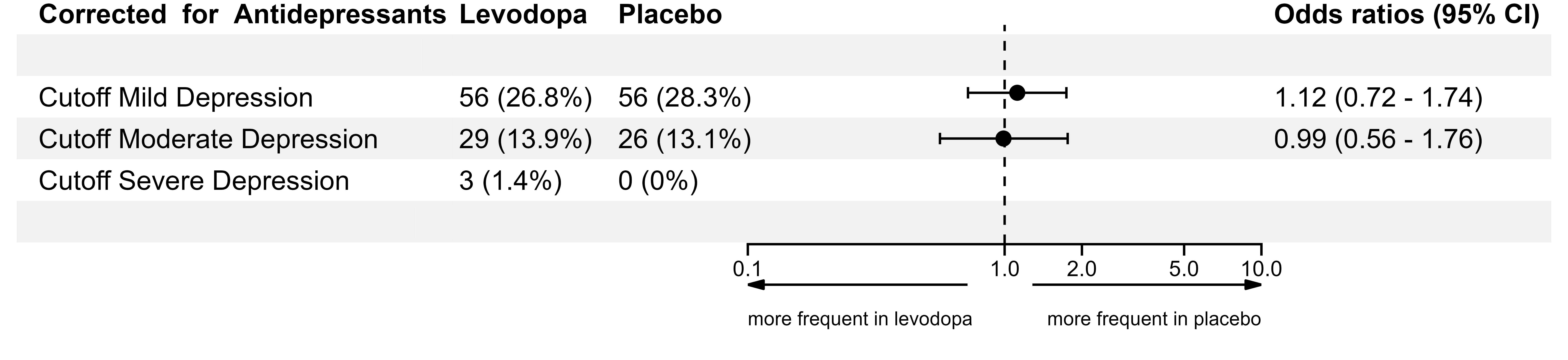
*Cutoff mild depression = T-score ≥55, cutoff moderate depression = T-score ≥60, cutoff severe depression = T-score ≥70.*

**Figure S2: Sensitivity analysis restricted to participants with antidepressant use during follow-up.**

The proportion of depression at three months is shown using different cutoff T-scores of the PROMIS short-form depression-4a among participants who received antidepressant medication at any time during follow-up, stratified by levodopa and placebo groups.


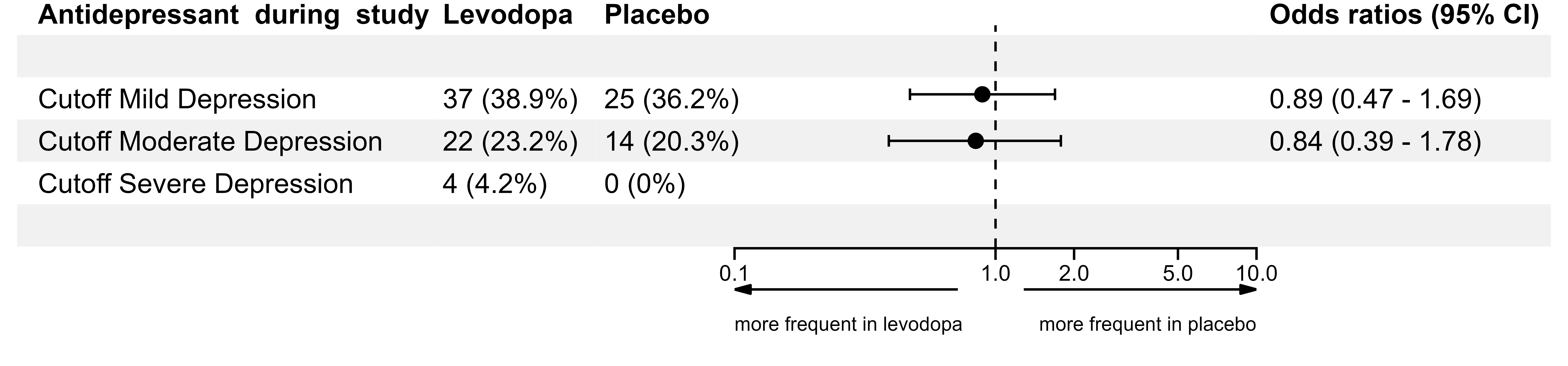
*Cutoff mild depression = T-score ≥55, cutoff moderate depression = T-score ≥60, cutoff severe depression = T-score ≥70.*

**Figure S3: Sensitivity analysis restricted to participants without antidepressant use during follow-up.**

The proportion of depression at three months is shown using different cutoff T-scores of the PROMIS short-form depression-4a among participants who did not receive antidepressant medication at any time during follow-up, stratified by levodopa and placebo groups.


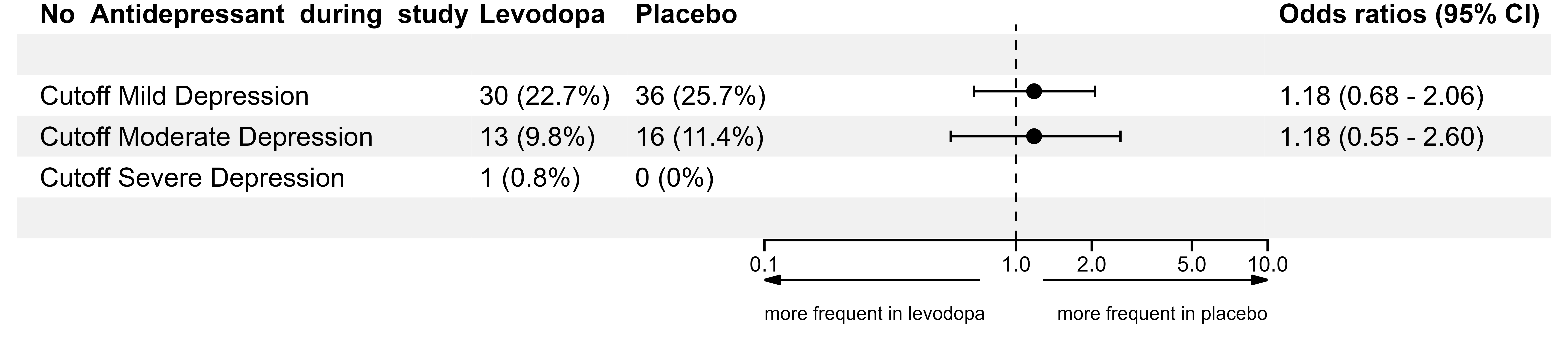
*Cutoff mild depression = T-score ≥55, cutoff moderate depression = T-score ≥60, cutoff severe depression = T-score ≥70.*

**Figure S4: Associations between PSD and participant characteristics.**

The median was chosen to convert each continuous numerical variable into a binary format, and univariate binary logistic regression models were applied to assess the associations.


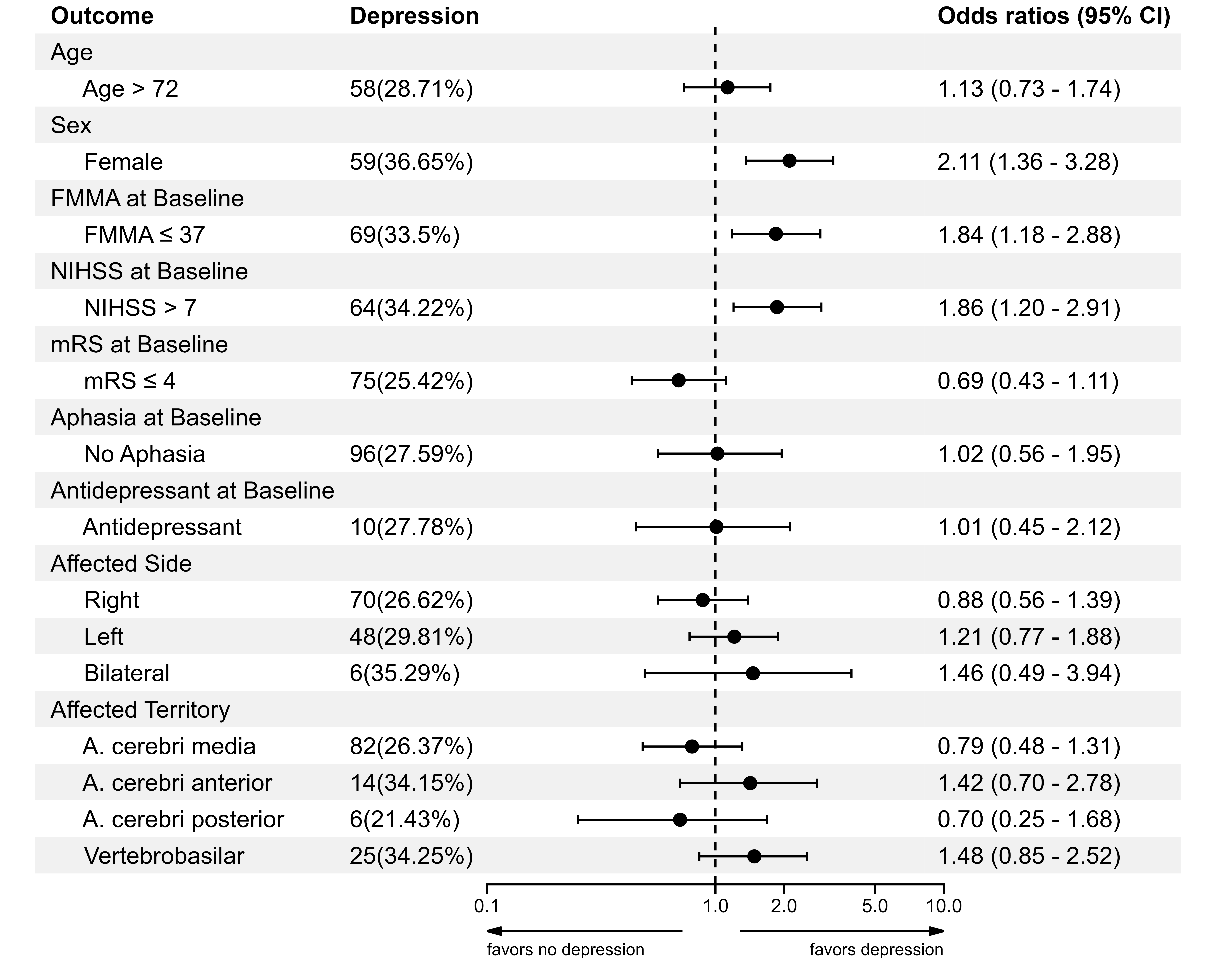
*FMMA = Fugl-Meyer Motor, mRS = modified Rankin Scale, NIHSS = National Institutes of Health Stroke Scale.*

**Figure S5****: Association between outcomes after three months and PSD.**

The median was chosen to convert each continuous numerical variable into a binary format, and univariate binary logistic regression models were applied to assess the associations.


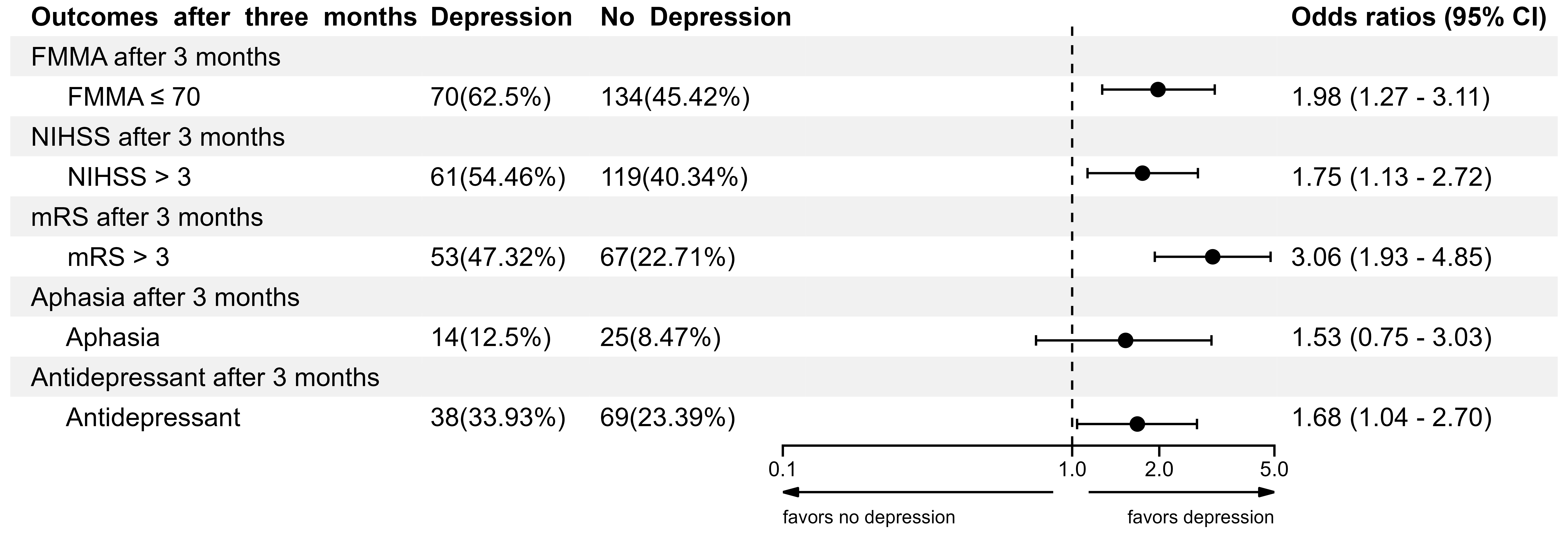
*FMMA = Fugl-Meyer Motor, NIHSS = National Institutes of Health Stroke Scale, mRS = modified Rankin Scale.*

**Figure S6: Use of antidepressants at baseline and during follow-up.**

Percentage of participants taking antidepressants each for the levodopa and placebo group, at baseline, after five weeks, and after three months.

*
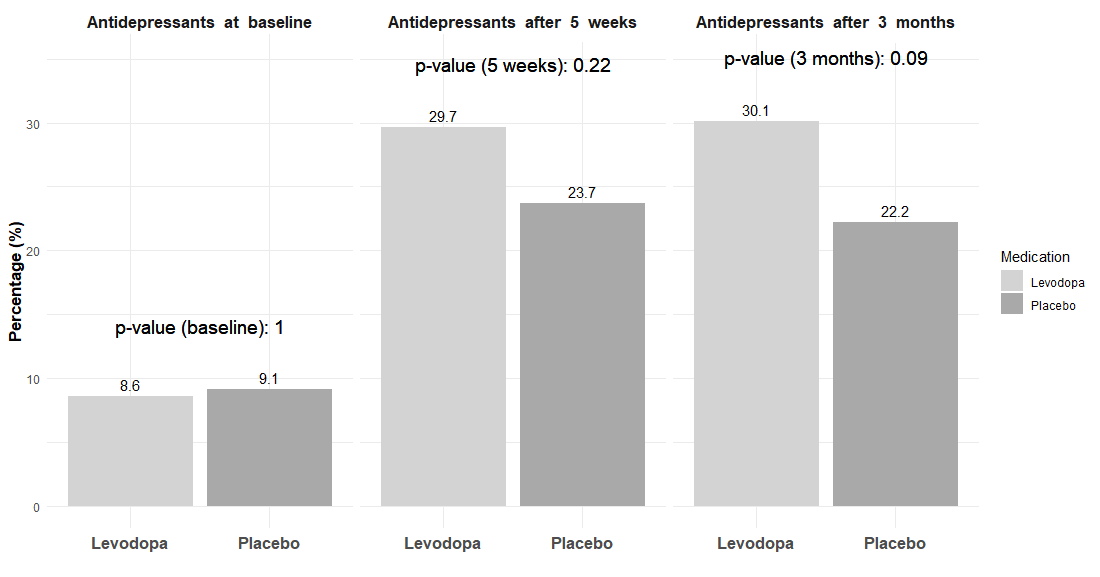
*
