# Supplementary material for: The impact of levodopa on post-stroke depression: the ESTREL-depression-study
Source: Eur Stroke J. 2026 Feb 17;11(2):aakag001. doi: 10.1093/esj/aakag001 (PMC12911922; doi:10.1093/esj/aakag001)
Supplement: aakag001_Supplement_2_Nonauthor_Collaborators [file aakag001_supplement_2_nonauthor_collaborators.pdf]

Supplementary Online Material: Nonauthor Collaborators
*First name, last name, and suffix (if applicable) are required and will appear in PubMed.

*Group Name(s): ESTREL Investigators							
*First Name and Middle Initial(s)	*Last Name	*Suffix (eg, Jr, III)	Academic Degrees	Institution	Location (city, state/province, country)	Role or Contribution, eg, chair, principal Investigator	Group (if more than 1 Group listed in the byline) and/or Subgroup (eg, Steering Committee)
Krassen	Nedeltchev			Kantonsspital Aarau		Local Principal Investigator	
Timo	Kahles			Kantonsspital Aarau		Sub-Investigator	
Sandra	Clarke			Kantonsspital Aarau		Study Nurse	
Catharina	Fritz-Rochner			Kantonsspital Baden		Study Nurse	
Alexander	Tarnutzer			Kantonsspital Baden		Local Principal Investigator	
Loric	Berney			Centre Hospitalier Universitaire Vaudois		Study Therapist	
Manuela	Buehrer			Centre Hospitalier Universitaire Vaudois		Study Nurse	
Karin	Diserens			Centre Hospitalier Universitaire Vaudois		Sub-Investigator	
Alexandra	Dos Reis Moreira			Centre Hospitalier Universitaire Vaudois		Study Therapist	
Grégoire	Eberle			Centre Hospitalier Universitaire Vaudois		Study Therapist	
Ashraf	Eskandari			Centre Hospitalier Universitaire Vaudois		Study Nurse	
Melissa	Esteves			Centre Hospitalier Universitaire Vaudois		Study Therapist	
Gaël	Hafner			Centre Hospitalier Universitaire Vaudois		Study Therapist	
Errikos	Maslias			Centre Hospitalier Universitaire Vaudois		Sub-Investigator	
Patrik	Michel			Centre Hospitalier Universitaire Vaudois		Local Principal Investigator	
Pierre-André	Rapin			Centre Hospitalier Universitaire Vaudois		Sub-Investigator	

Supplementary Online Material: Nonauthor Collaborators
*First name, last name, and suffix (if applicable) are required and will appear in PubMed.

*First Name and Middle Initial(s)	*Last Name	*Suffix (eg, Jr, III)	Academic Degrees	Institution	Location (city, state/province, country)	Role or Contribution, eg, chair, principal Investigator	Group (if more than 1 Group listed in the byline) and/or Subgroup (eg, Steering Committee)
Suzette	Remillard			Centre Hospitalier Universitaire Vaudois		Study Nurse	
Alexander	Salerno			Centre Hospitalier Universitaire Vaudois		Sub-Investigator	
Davide	Strambo			Centre Hospitalier Universitaire Vaudois		Sub-Investigator	
Guillaume	Thevoz			Centre Hospitalier Universitaire Vaudois		Sub-Investigator	
Katrien	Van Den Keybus Deglon			Centre Hospitalier Universitaire Vaudois		Study Therapist	
Elodie	Cottier			Hôpital Fribourgeois		Study Therapist	
Aline	Duc			Hôpital Fribourgeois		Study Therapist	
Sandrine	Foucras			Hôpital Fribourgeois		Study Nurse	
Friedrich	Medlin			Hôpital Fribourgeois		Local Principle Investigator	
Martine	Rime			Hôpital Fribourgeois		Study Nurse	
Romain	Stohr			Hôpital Fribourgeois		Study Therapist	
Sabine	Spring			Hôpital Fribourgeois Meyriez-Murten		Study Nurse	
Barbara	Bleuler			Klinik Hirslanden		Study Therapist	
Marion	Huser			Klinik Hirslanden		Study Therapist	
Joelle	Keller			Klinik Hirslanden		Study Nurse	
Fabienne	Maibach			Klinik Hirslanden		Sub-Investigator	
Maja	Müller			Klinik Hirslanden		Study Nurse	
Nils	Peters			Klinik Hirslanden		Local Principle Investigator	
Steven	Rieger			Klinik Hirslanden		Study Therapist	
Janine	Schär			Klinik Hirslanden		Sub-Investigator	
Shadi	Taheri			Klinik Hirslanden		Sub-Investigator	
Carina	Wehrli			Klinik Hirslanden		Sub-Investigator	
Sereina	Zwissler			Klinik Hirslanden		Study Therapist	

Supplementary Online Material: Nonauthor Collaborators
*First name, last name, and suffix (if applicable) are required and will appear in PubMed.

*First Name and Middle Initial(s)	*Last Name	*Suffix (eg, Jr, III)	Academic Degrees	Institution	Location (city, state/province, country)	Role or Contribution, eg, chair, principal Investigator	Group (if more than 1 Group listed in the byline) and/or Subgroup (eg, Steering Committee)
Marcel	Arnold			Inselspital, Universitätsspital Bern		Local Co-Principle Investigator	
Elias	Auer			Inselspital, Universitätsspital Bern		Sub-Investigator	
Morin	Beyeler			Inselspital, Universitätsspital Bern		Sub-Investigator	
Anna	Boronylo			Inselspital, Universitätsspital Bern		Sub-Investigator	
Yvonne	Brückner			Inselspital, Universitätsspital Bern		Study Nurse	
Christine	Brülisauer			Inselspital, Universitätsspital Bern		Study Coordinator	
Leander	Clénin			Inselspital, Universitätsspital Bern		Sub-Investigator	
Boudewijn	Drop			Inselspital, Universitätsspital Bern		Sub-Investigator	
Julie	Hanke			Inselspital, Universitätsspital Bern		Sub-Investigator	
Eveline	Kaufmann			Inselspital, Universitätsspital Bern		Study Therapist	
Marianne	Kormann			Inselspital, Universitätsspital Bern		Study Coordinator	
Martin	Lange			Inselspital, Universitätsspital Bern		Study Nurse	
Christina	Leistner			Inselspital, Universitätsspital Bern		Sub-Investigator	
Basel	Maamari			Inselspital, Universitätsspital Bern		Sub-Investigator	
Liselotte	McEvoy			Inselspital, Universitätsspital Bern		Study Nurse	
Madlaine	Müller			Inselspital, Universitätsspital Bern		Sub-Investigator	
René	Müri			Inselspital, Universitätsspital Bern		Local Principle Investigator	
Janis Patricia	Rauch			Inselspital, Universitätsspital Bern		Sub-Investigator	
Jasmin	Schlatter			Inselspital, Universitätsspital Bern		Study Therapist	
Adrian	Scutelnic			Inselspital, Universitätsspital Bern		Sub-Investigator	
Bernhard Matthias	Siepen			Inselspital, Universitätsspital Bern		Sub-Investigator	
Norbert	Silimon			Inselspital, Universitätsspital Bern		Sub-Investigator	
Vasileios	Tentolouris Piperas			Inselspital, Universitätsspital Bern		Sub-Investigator	
Jan	Vynckier			Inselspital, Universitätsspital Bern		Sub-Investigator	
Johanna	Weghorn			Inselspital, Universitätsspital Bern		Study Therapist	
Valerian	Altersberger			Universitätsspital Basel		Medical Network Advisor	
Nikolaos, Symeon	Avramiotis			Universitätsspital Basel		Sub-Investigator	

Supplementary Online Material: Nonauthor Collaborators
*First name, last name, and suffix (if applicable) are required and will appear in PubMed.

*First Name and Middle Initial(s)	*Last Name	*Suffix (eg, Jr, III)	Academic Degrees	Institution	Location (city, state/province, country)	Role or Contribution, eg, chair, principal Investigator	Group (if more than 1 Group listed in the byline) and/or Subgroup (eg, Steering Committee)
Lea	Barone			Universitätsspital Basel		Sub-Investigator	
Lukas	Boos			Universitätsspital Basel		Sub-Investigator	
Eveline	Brunner			Universitätsspital Basel		Sub-Investigator	
Gian Marco	De Marchis			Universitätsspital Basel		Sub-Investigator	
Stefan	Engelter			Universitätsspital Basel		Sponsor Investigator	
Fabina	Engelter			Universitätsspital Basel		Student	
Sophia	Engelter			Universitätsspital Basel		Student	
Sandro	Fischer			Universitätsspital Basel		Sub-Investigator	
Joachim	Fladt			Universitätsspital Basel		Sub-Investigator	
Henrik	Gensicke			Universitätsspital Basel		Sub-Investigator	
Jasmine	Jost			Universitätsspital Basel		Sub-Investigator	
Mira	Katan			Universitätsspital Basel		Sub-Investigator	
Josefin	Kaufmann			Universitätsspital Basel		Medical Network Advisor	
Wilma	Kamerbeek Verhagen			Universitätsspital Basel		Data manager	
Martin	Lucht			Universitätsspital Basel		Study Coordination	
Philippe	Lyrer			Universitätsspital Basel		Local Principal Investigator	
Yasmin	Liechti			Universitätsspital Basel		Study Nurse	
Marina	Maurer			Universitätsspital Basel		Study Coordination	
Louisa	Meya			Universitätsspital Basel		Sub-Investigator	
Daniela	Minio			Universitätsspital Basel		Study Nurse	
Lukas	Nussbaum			Universitätsspital Basel		Sub-Investigator	
Larissa	Nussbaumer			Universitätsspital Basel		Sub-Investigator	
Nils	Peters			Universitätsspital Basel		Sub-Investigator	
Ines	Piot			Universitätsspital Basel		Sub-Investigator	
Alexandros	Polymeris			Universitätsspital Basel		Medical Network Advisor	
Flavia	Ravanelli			Universitätsspital Basel		Sub-Investigator	
Mirjam	Sauter			Universitätsspital Basel		Sub-Investigator	
Iris	Schneider			Universitätsspital Basel		Study Nurse	
Christopher	Tränka			Universitätsspital Basel		Medical Network Advisor	

Supplementary Online Material: Nonauthor Collaborators
*First name, last name, and suffix (if applicable) are required and will appear in PubMed.

*First Name and Middle Initial(s)	*Last Name	*Suffix (eg, Jr, III)	Academic Degrees	Institution	Location (city, state/province, country)	Role or Contribution, eg, chair, principal Investigator	Group (if more than 1 Group listed in the byline) and/or Subgroup (eg, Steering Committee)
Simon	Trüssel			Universitätsspital Basel		Sub-Investigator	
Benjamin	Wagner			Universitätsspital Basel		Sub-Investigator	
Martina	Wiegert			Universitätsspital Basel		Study Coordination, Study Nurse	
Karin	Wiesner			Universitätsspital Basel		Study PhysioTherapist	
Céline	Zbinden			Universitätsspital Basel		Sub-Investigator	
Annaelle	Zietz			Universitätsspital Basel		Medical Network Advisor	
Meret	Branscheidt			Universitätsspital Zürich		Sub-Investigator	
Ioannis	Georgiou			Universitätsspital Zürich		Sub-Investigator	
Christoph	Globas			Universitätsspital Zürich		Sub-Investigator	
Marcellina	Haeberlin			Universitätsspital Zürich		Sub-Investigator	
Jeremia	Held			Universitätsspital Zürich		Sub-Investigator, Study Coordinator, Study Therapist	
Eva	Heusi-Thürlimann			Universitätsspital Zürich		Study Therapist	
Mira	Katan			Universitätsspital Zürich		Sub-Investigator	
Aurelia	Lehmann			Universitätsspital Zürich		Study Therapist	
Andreas	Luft			Universitätsspital Zürich		Local Principle Investigator	
Theodor	Pipping			Universitätsspital Zürich		Sub-Investigator	
Johannes	Pohl			Universitätsspital Zürich		Study Therapist	
Todor	Popov			Universitätsspital Zürich		Sub-Investigator	
Yannik	Rottenberger			Universitätsspital Zürich		Study Coordinator, Study Therapist	
Anne	Schwarz			Universitätsspital Zürich		Study Therapist	
Juliane	Schweizer			Universitätsspital Zürich		Sub-Investigator	
Ursula	Surbeck			Universitätsspital Zürich		Study Therapist	
Jannie	van Duinen			Universitätsspital Zürich		Study Coordinator	
Janne	Veerbeek			Universitätsspital Zürich		Study Therapist	
Susanne	Wegener			Universitätsspital Zürich		Sub-Investigator	

Supplementary Online Material: Nonauthor Collaborators
*First name, last name, and suffix (if applicable) are required and will appear in PubMed.

*First Name and Middle Initial(s)	*Last Name	*Suffix (eg, Jr, III)	Academic Degrees	Institution	Location (city, state/province, country)	Role or Contribution, eg, chair, principal Investigator	Group (if more than 1 Group listed in the byline) and/or Subgroup (eg, Steering Committee)
Rolf	Sturzenegger			Kantonsspital Graubünden		Local Principal Investigator	
Sylvan	Albert			Kantonsspital Graubünden		Sub-Investigator	
Christa	Caflisch			Kantonsspital Graubünden		Study Therapist	
Markus	Baumgaertner			Kantonsspital Münsterlingen		Local Principal Investigator	
Svetlana	Politz			Kantonsspital Münsterlingen		Study Coordinator	
Ludwig	Schelosky			Kantonsspital Münsterlingen		Local Principal Investigator	
Vjosa	Iljazi			Kantonsspital St. Gallen		Study Nurse	
Georg	Kägi			Kantonsspital St. Gallen		Local Principal Investigator	
Anna	Müller			Kantonsspital St. Gallen		Study Nurse	
Jochen	Vehoff			Kantonsspital St. Gallen		Sub-Investigator	
Valerian	Altersberger			Felix Platter Spital		Medical Network Advisor	
Nikolaos, Symeon	Avramiotis			Felix Platter Spital		Sub-Investigator	
Lea	Barone			Felix Platter Spital		Sub-Investigator	
Lukas	Boos			Felix Platter Spital		Sub-Investigator	
Eveline	Brunner			Felix Platter Spital		Sub-Investigator	
Stefan	Engelter			Felix Platter Spital		Sponsor-Investigator	
Fabian	Engelter			Felix Platter Spital		Student	
Sophia	Engelter			Felix Platter Spital		Student	
Sandro	Fischer			Felix Platter Spital		Sub-Investigator	
Matthias	Frank			Felix Platter Spital		Sub-Investigator	
Henrik	Gensicke			Felix Platter Spital		Local Principal Investigator	
Jasmine	Jost			Felix Platter Spital		Sub-Investigator	
Josefin	Kaufmann			Felix Platter Spital		Medical Network Advisor	
Claire	Liniger			Felix Platter Spital		PhysioTherapist	
Yasmin	Liechti			Felix Platter Spital		Study Nurse	
Martin	Lucht			Felix Platter Spital		Study Coordination	

Supplementary Online Material: Nonauthor Collaborators
*First name, last name, and suffix (if applicable) are required and will appear in PubMed.

*First Name and Middle Initial(s)	*Last Name	*Suffix (eg, Jr, III)	Academic Degrees	Institution	Location (city, state/province, country)	Role or Contribution, eg, chair, principal Investigator	Group (if more than 1 Group listed in the byline) and/or Subgroup (eg, Steering Committee)
Louisa	Meya			Felix Platter Spital		Sub-Investigator	
Daniela	Minio			Felix Platter Spital		Study Nurse	
Lukas	Nussbaum			Felix Platter Spital		Sub-Investigator	
Larissa	Nussbaumer			Felix Platter Spital		Sub-Investigator	
Vera	Paltzer			Felix Platter Spital		Study Therapist	
Nils	Peters			Felix Platter Spital		Principal Investigator	
Ines	Piot			Felix Platter Spital		Sub-Investigator	
Alexandros	Polymeris			Felix Platter Spital		Medical Network Advisor	
Flavia	Ravanelli			Felix Platter Spital		Sub-Investigator	
Mirjam	Sauter			Felix Platter Spital		Sub-Investigator	
Christopher	Tränka			Felix Platter Spital		Medical Network Advisor	
Simon	Trüssel			Felix Platter Spital		Sub-Investigator	
Annick	Voeste			Felix Platter Spital		Study Therapist	
Martina	Wiegert			Felix Platter Spital		Study Coordination, Study Nurse	
Karin	Wiesner			Felix Platter Spital		Study PhysioTherapist	
Céline	Zbinden			Felix Platter Spital		Sub-Investigator	
Annaelle	Zietz			Felix Platter Spital		Medical Network Advisor	
Frank	Behrendt			Reha Rheinfelden		Study-Investigator	
Leo	Bonati			Reha Rheinfelden		Local Principle Investigator	
Heiner	Brunnschweiler			Reha Rheinfelden		Sub-Investigator	
Thierry	Ettlin			Reha Rheinfelden		Local Principle Investigator	
Szabina	Gäumann			Reha Rheinfelden		Study Nurse, Study Therapist	
Sarah	Hermann			Reha Rheinfelden		Study Nurse, Study Therapist	
Corina	Schuster-Amft			Reha Rheinfelden		Sub-Investigator	

Supplementary Online Material: Nonauthor Collaborators
*First name, last name, and suffix (if applicable) are required and will appear in PubMed.

*First Name and Middle Initial(s)	*Last Name	*Suffix (eg, Jr, III)	Academic Degrees	Institution	Location (city, state/province, country)	Role or Contribution, eg, chair, principal Investigator	Group (if more than 1 Group listed in the byline) and/or Subgroup (eg, Steering Committee)
Zorica	Suica			Reha Rheinfelden		Study Nurse, Study Therapist	
Niklaus	Urscheler			Reha Rheinfelden		Sub-Investigator	
Pharmacy of the rehabilitation centre Reha Rheinfelden				Reha Rheinfelden		Study Therapist	
Marco	Eugster			Reha Valens		Study Therapist	
Carmen	Lienert			Reha Valens		Local Principal Investigator	
Roman	Gonzenbach			Reha Valens		Local Principal Investigator	
Yuka	Hasegawa			Reha Valens		Study Therapist	
Manuela	Marugg			Reha Valens		Study Nurse	
Christine	Meier Khan			Reha Valens		Study Therapist	
Veit	Mylius			Reha Valens		Sub-Investigator	
Heike	Rosemeyer			Reha Valens		Study Therapist	
